# BCAS1-positive immature oligodendrocytes are affected by the α-synuclein-induced pathology of multiple system atrophy

**DOI:** 10.1186/s40478-020-00997-4

**Published:** 2020-07-29

**Authors:** Seiji Kaji, Takakuni Maki, Jun Ueda, Tomoyuki Ishimoto, Yutaka Inoue, Ken Yasuda, Masanori Sawamura, Rie Hikawa, Takashi Ayaki, Hodaka Yamakado, Ryosuke Takahashi

**Affiliations:** Department of Neurology, Graduate School of Medicine, Kyoto University, 54 Shogoin-Kawahara-cho, Sakyo-ku, Kyoto, 606-8397 Japan

**Keywords:** α-Synuclein, Breast cancer amplified sequence 1, Glial cytoplasmic inclusions, Maturation, Multiple system atrophy, Myelination, Oligodendrocytes, Oligodendrocyte precursor cells

## Abstract

Multiple system atrophy (MSA) is pathologically characterized by the presence of fibrillar α-synuclein-immunoreactive inclusions in oligodendrocytes. Although the myelinating process of oligodendrocytes can be observed in adult human brains, little is known regarding the presence of α-synuclein pathology in immature oligodendrocytes and how their maturation and myelination are affected in MSA brains. Recently, breast carcinoma amplified sequence 1 (BCAS1) has been found to be specifically expressed in immature oligodendrocytes undergoing maturation and myelination. Here, we analyzed the altered dynamics of oligodendroglial maturation in both MSA brains and primary oligodendroglial cell cultures which were incubated with α-synuclein pre-formed fibrils. The numbers of BCAS1-expressing oligodendrocytes that displayed a matured morphology negatively correlated with the density of pathological inclusions in MSA brains but not with that in Parkinson’s disease and diffuse Lewy body disease. In addition, a portion of the BCAS1-expressing oligodendrocyte population showed cytoplasmic inclusions, which were labeled with antibodies against phosphorylated α-synuclein and cleaved caspase-9. Further in vitro examination indicated that the α-synuclein pre-formed fibrils induced cytoplasmic inclusions in the majority of BCAS1-expressing oligodendrocytes. In contrast, the majority of BCAS1-non-expressing mature oligodendrocytes did not develop inclusions on day 4 after maturation induction. Furthermore, exposure of α-synuclein pre-formed fibrils in the BCAS1-positive phase caused a reduction in oligodendroglial cell viability. Our results indicated that oligodendroglial maturation and myelination are impaired in the BCAS1-positive phase of MSA brains, which may lead to the insufficient replacement of defective oligodendrocytes. In vitro, the high susceptibility of BCAS1-expressing primary oligodendrocytes to the extracellular α-synuclein pre-formed fibrils suggests the involvement of insufficient oligodendroglial maturation in MSA disease progression and support the hypothesis that the BCAS1-positive oligodendrocyte lineage cells are prone to take up aggregated α-synuclein in vivo.

## Introduction

The accumulation of misfolded α-synuclein (α-syn) within oligodendrocytes (OLGs), known as glial cytoplasmic inclusions (GCIs), is considered to be responsible for both oligodendroglial dysfunction and neurodegeneration, which can trigger the onset of symptoms in multiple system atrophy (MSA) [21]. As part of the repair mechanism against brain injury, OLGs can be regenerated through the maturation of OLG precursor cells (OPCs), which are a type of glial cell distributed diffusely within the adult human brain [3]. However, several in vitro experiments have reported the insufficient maturation of OPCs due to the presence of intracellular α-syn, raising the possibility that oligodendroglial dysfunction in MSA brains is not compensated by OLG regeneration [5, 7, 8].

**Fig. 1:**
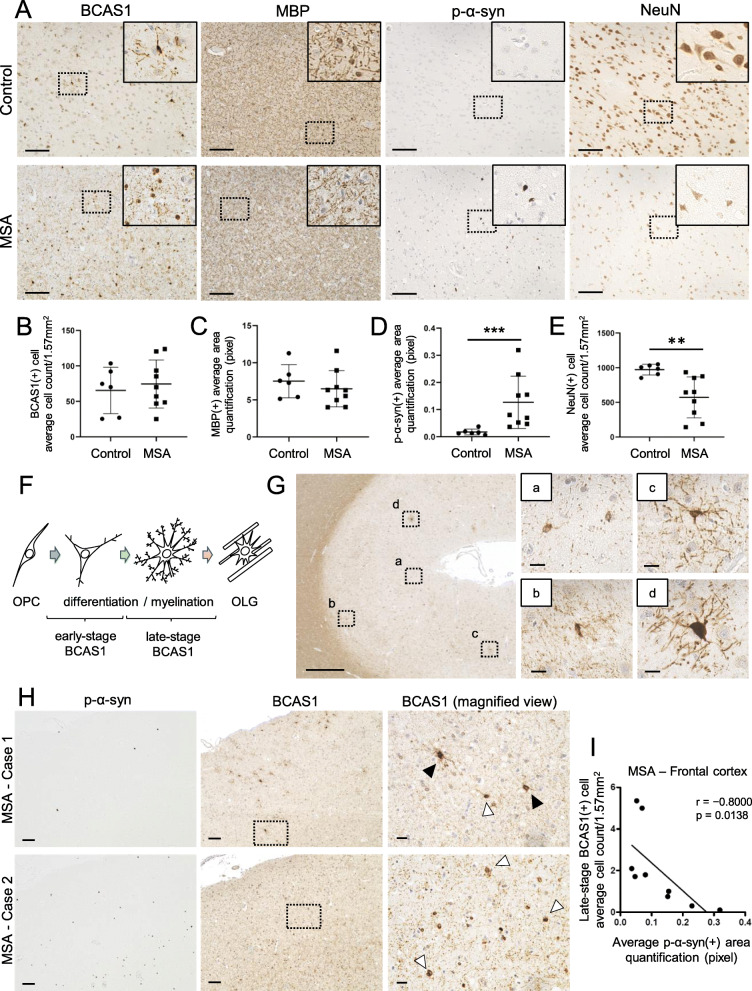
The differentiation of BCAS1(+) cells was affected by p-α-syn-immunoreactive inclusions in the frontal cortices of MSA brains. **a** Serial sections of brain tissues from the frontal cortices of an aged non-neurodegenerative disease control (upper) and an MSA patient (lower) which were immunostained with antibodies against BCAS1, MBP, p-α-syn, and NeuN. Scale bar = 100 μm. The regions marked by dotted squares are magnified in the right upper corners. The magnified images of BCAS1 staining show altered morphology of BCAS1(+) cells in the MSA case. Images of control and MSA cases are provided from case 21 and case 2 (Additional file 1: Table S1), respectively. **b**, **e** Quantification of BCAS1(+) cell counts (**b**), MBP(+) cell counts (**c**), p-α-syn(+) area (**d**), and NeuN(+) cell counts (**e**) in control and MSA brains. Mean ± SEM; control, *N* = 6, MSA, *N* = 9; Mann–Whitney, p** < 0.01, p*** < 0.001. **f** Illustration of oligodendroglial differentiation showing the morphological classification of early-stage and late-stage BCAS1(+) cells. **g** Immunostaining of an aged non-neurodegenerative disease control (case 21, Additional file 1: Table S1) with the anti-BCAS1 antibody showing representative images of early-stage (a, b) and late-stage (c, d) BCAS1(+) cells in the frontal cortex. Scale bar = 500 μm (low-power field, left) and 20 μm (high-power field, right). (H) Immunostaining of the serial sections from the frontal cortices of two different MSA cases (cases 1 and 2, Additional file 1: Table S1) showing the morphological variety of BCAS1(+) cells and their association with the density of the p-α-syn(+) area. Black arrowheads indicate late-stage BCAS1(+) cells, whereas white arrowheads indicate early-stage BCAS1(+) cells. Case 1 with sparse cortical p-α-syn pathology shows multi-processed mature BCAS1(+) cells. In contrast, case 2 with notable p-α-syn pathology shows round BCAS1(+) cells with few processes. Scale bar = 100 μm (low-power field, left and middle) and 20 μm (high-power field, right). (I) Linear regression analysis showing a negative correlation between the number of late-stage BCAS1(+) cells and the density of p-α-syn(+) in the frontal cortices of MSA patients (*N* = 9, Spearman r = − 0.8000, *p* = 0.0138)

Very few post-mortem investigations have focused on the involvement of immature OLGs in the disease pathomechanisms of MSA. Although some reports have indicated the presence of α-syn-immunopositive inclusions in OPCs, it remains uncertain whether α-syn pathology affects immature OLGs in the brain of MSA patients [8, 13]. In addition, various obstacles exist with regard to the post-mortem investigation of immature OLGs, including the low sensitivity of antibodies against OPC-specific antigens such as NG2 and PDGFRα [19]. However, the expression of the recently characterized oligodendroglial cell marker, breast-carcinoma amplified sequence 1 (BCAS1), specifically identifies immature OLGs, which are transiently present during the active phase of OLG generation and myelination/remyelination in both rodent and human brains (Fig. 1f) [6, 16]. BCAS1-expressing (BCAS1(+)) cells represent a distinct oligodendroglial cell population that can be distinguished by the markers for OPCs and mature OLGs. In fact, while BCAS1(+) cells express OLIG2 and SOX10, human data suggest that they show limited immunolabeling with other oligodendroglial cell markers, such as neural/glial antigen 2 (NG2) (3.5%), tubulin polymerization promoting protein (TPPP/p25α) (0.6%), CC1 (0%), proteolipid protein (PLP) (0%), and myelin basic protein (MBP) (7.5%) [6]. Immunohistochemically, these cells typically display multiple symmetrically radiating processes or segments aligned in parallel. Although the density of BCAS1(+) cells is the highest in the first year of postnatal white matter development, they are constantly observed throughout the adult life, particularly in the frontal cortices, possibly reflecting the presence of adaptive myelination [6]. In addition, BCAS1(+) cells are known to be increased within the remyelinating areas of multiple sclerosis, suggesting that a portion of the BCAS1(+) cell population is engaged in the active regeneration of OLGs [6].

In the present study, we sought to reveal the pathological impact of α-syn on oligodendroglial maturation in MSA brains, by identifying the BCAS1(+) cell population and confirming the presence of intracellular α-syn-immunoreactive inclusions in these immature OLGs. To our knowledge, this is the first report to histopathologically describe the altered dynamics of OLG regeneration in MSA brains.

## Materials and methods

### Human brain tissue

**Fig. 2:**
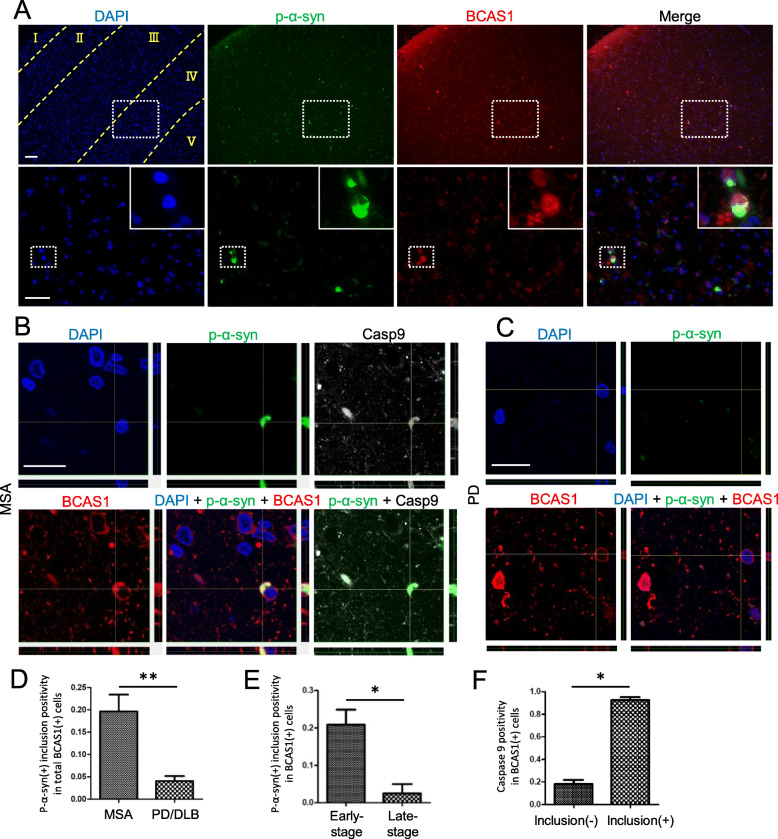
p-α-Syn-immunoreactive inclusions were present in the cytoplasm of BCAS1(+) cells in MSA brains. **a** Immunofluorescence staining of an MSA patient brain showing p-α-syn-immunoreactive inclusions in the deep layer of the frontal cortex (low-power field images, upper) and the presence of inclusions within the BCAS1(+) cells (high-power field images, lower). The borders between cortical layers were defined by NeuN and Kluver–Barrera (data not shown) staining and are indicated as yellow dotted lines for DAPI staining. The areas marked by white dotted lines in the upper images are magnified in the lower images, where further magnified images of inclusion-positive cells are displayed in the top right corners. Scale bars = 100 μm (upper) and 50 μm (lower). The images are provided from case 2 (Additional file 1: Table S1). **b** Confocal images of BCAS1(+) cells in the frontal cortex of an MSA patient showing the cytoplasmic inclusions, which were immunoreactive to antibodies against p-α-syn and cleaved caspase-9. Scale bar = 20 μm. The images are provided from case 9 (Additional file 1: Table S1). **c** Confocal images of BCAS1(+) cells in the frontal cortex of a PD patient showing no intracellular p-α-syn immunoreactivity. Scale bar = 20 μm. The images are provided from case 14 (Additional file 1: Table S1). **d** The frequencies of p-α-syn-immunoreactive inclusions in the BCAS1(+) cells of MSA brains and PD/DLB brains (frontal cortex) were compared. PD/DLB, *N* = 7, MSA, *N* = 4; Mann–Whitney, p** < 0.01. **e** The frequencies of p-α-syn-immunoreactive inclusions between early-stage and late-stage BCAS1(+) cells of the MSA brains were compared. *N* = 4, respectively; Mann–Whitney, p* < 0.05. **f** The frequencies of cleaved caspase-9 immunoreactivity were compared between BCAS1(+) cells with and without p-α-syn-immunoreactive inclusions. *N* = 4, respectively; Mann–Whitney, p* < 0.05

For immunohistochemical analysis, formalin-fixed, paraffin-embedded 6-μm-thick sections from the frontal lobe of the cerebral cortex, pons, cerebellum, and putamen of patients with MSA, Parkinson’s disease (PD)/diffuse Lewy body disease (DLB), and non-neurodegenerative disease controls were deparaffinized and immunostained (see Additional file 1: Table S1 and Additional file 2: Fig. S1 for detailed description of the clinical information and studied regions in each case). Cases with more than 1-month duration of premortal ventilation or a history of sustained generalized seizures were excluded from our analysis. Analysis of oligodendroglial cell populations and p-α-syn pathology in the frontal cortex as well as that of the white matter in the corresponding frontal lobe were performed in nine MSA cases, nine PD/DLB cases, and six controls (Fig. 1; Additional file 3: Fig. S2A–E). BCAS1 and phosphorylated α-syn staining of the putamen, pons, and cerebellum was conducted in four MSA cases and two controls (Additional file 3: Fig. S2A). For the detection of cytoplasmic inclusions using immunofluorescence staining, cases with a cortical α-syn-pathology score of “stage 2 (moderate)” or greater (MSA; *N* = 4, PD/DLB; *N* = 7), which is the criteria used for the diagnosis of DLB, were used to compare the emergence of α-syn-pathology in BCAS1(+) cells (Fig. 2; Additional file 3: Fig. S2F–I) [14]. The detection of such inclusions in BCAS1(+) cells in the pons was investigated in one MSA case (case 10; Additional file 1: Table S1; Additional file 3: Fig. S2J). All procedures involving the use of human materials were performed in accordance with the ethical guidelines set by Kyoto University.

### Immunohistochemistry

After heat-induced antigen retrieval with citrate buffer (pH 6.0), human brain sections were incubated overnight at 4 °C with primary antibodies against phosphorylated α-syn (1:1000, Abcam, ab51253), BCAS1 (1:1000, Thermo Fisher, PA5–20904), MBP (1:1000, Thermo Fisher, MA1–10837), and NeuN (1:1000, Merck Millipore, ABN78). For visualization with secondary antibodies, Histofine (Nichirei Bioscience) was used for diaminobenzidine staining. In addition, Kluver–Barrera staining of each section was used to distinguish the cortical areas from white matter. For MBP- and phosphorylated α-syn-immunoreactive area quantification and total BCAS1(+) and NeuN(+) cell count, average values per each 1.57 mm^2^ visual field were calculated using Image J analysis, with 10–14 images captured from each case. Late-stage BCAS1(+) cells were defined morphologically as BCAS1(+) cells with four or more processes and included cells with myelinating morphology. Early BCAS1(+) cells were defined as those with three or less processes.

### Double-staining of DAB and alkaline phosphatase (AP)

For double staining of DAB and AP, the following primary antibodies were used: NaBC1, 1:1000, Santa Cruz, sc-136,342; BCAS1, 1:1000, Thermo Fisher, PA5–20904; NG2, 1:50, Millipore, AB5320; and APC, 1:50, OP80, CalBio. Antigen retrieval was performed by autoclaving for 20 min at 120 °C using the Histofine deparaffinizing antigen retrieval buffer, pH 6 (Cat No. 415281; Nichirei, Tokyo, Japan) followed by overnight incubation at 4 °C with the first primary antibodies in phosphate-buffered saline (PBS) containing 3% bovine serum albumin. The samples were then washed three times with 0.01 M PBS with Triton X (PBS-T). Subsequently, the sections were incubated with peroxidase-conjugated anti-mouse IgG (Histofine Simple Stain Max-PO (M), Nichirei, Tokyo, Japan) for 30 min at 37 °C. Reaction products were visualized by immersing the slides in DAB solution (DAB Substrate Kit SK 4100, Vector Laboratories, Burlingame, CA, USA) for 3 min. After the sections were washed with deionized water, the second primary antibody was applied and incubated overnight at 4 °C. The sections were then washed three times with PBS-T before incubating with alkaline phosphatase-conjugated anti-rabbit IgG antibody (Histofine Simple Stain AP(R), Nichirei, Tokyo, Japan) for 30 min at 37 °C. The sections were again washed three times with 0.01 M PBS-T. Finally, the sections were visualized using Fast Red Substrate Kit (Cat No. 415261; Nichirei, Tokyo, Japan) according to the manufacturer’s instructions.

### Immunofluorescence staining

For human samples, antigen retrieval was performed as described above, and sections were incubated overnight at 4 °C with primary antibodies against phosphorylated α-syn (1:200, Wako, 015–25,191), BCAS1 (1:200, Thermo Fisher, PA5–20904), cleaved caspase-9 (h331) (1:200, Santa Cruz, sc-22,182), MBP (1:200, Thermo Fisher, MA1–10837), and α-syn (1:200, BD Biosciences, 610,787). Samples were subsequently incubated with donkey-derived secondary antibodies (Alexa Fluor 488, 594, and 647, 1:200, Thermo Fisher) for 1 h at room temperature. Sections were then covered with VECTASHIELD mounting medium (Vector Laboratories, H-1200) with DAPI. For thioflavin S assessment, sections were incubated with 20 μM thioflavin S (Sigma Aldrich, T1892) in distilled water for 20 min at room temperature before mounting. Images were obtained using an Olympus Fluoview FV1000 confocal microscope (Olympus), with image analysis and 3-D surface reconstruction performed by FV10-ASW software (Olympus). Sections were imaged at 0.46 μm in the z dimension. For the quantitative evaluation of α-syn immunoreactivity within the BCAS1(+) cells, a total of 60–120 cells, which included 5–10 late-stage BCAS1(+) cells per each case, were analyzed three-dimensionally.

For the immunofluorescence analysis of primary rat cell and slice cultures, samples were fixed with 4% PFA for 15 min. After a 10-min incubation with PBS/0.1%Tween, the samples were incubated overnight at 4 °C with primary antibodies against BCAS1 (1:200, Bioss antibodies, bs-11462R), rat and human α-syn (1:200, BD Biosciences, 610,787), human α-syn (1:200, Thermo Fisher Scientific, 180,215), PDGFRα (1:200, R&D systems, AF1062), MBP (1:200, Thermo Fisher, MA1–10837), NG2 (1:200, Millipore, AB5320), CC1 (APC) (Calbiochem, OP80), Olig2 (1:200, R&D, AF2418), and cleaved caspase-9 (Asp330) (1:200, Affinity, AF5244). Visualization with secondary antibodies and thioflavin S staining were conducted as described above. The intracellular immunoreactivity of α-syn and its colocalization with the LysoTracker probe were confirmed by confocal microscopy as described above. The quantitative assessment of intracellular inclusions in OLG lineage cells was performed with BZ-X710 (Keyence). The average counts of the cells containing thioflavin S-positive inclusions were obtained from four visual fields captured at a 20 × magnification, and compared between PDGFRα(+)/BCAS1(−) and PDGFRα(+)/BCAS1(+) cells as well as between BCAS1(+)/MBP(+) and BCAS1(−)/MBP(+) cells.

### Primary OLG lineage cell culture

OPCs were prepared as previously described [8, 10]. Briefly, the cerebral cortices from 1- to 2- day-old Sprague Dawley rats (Shimizu Laboratory Supplies Co., Ltd) were dissected, minced, and digested. Dissociated cells were plated in poly-D-lysine-coated 75 cm^2^ flasks, and maintained in Dulbecco’s Modified Eagle’s Medium (DMEM) containing 20% heat-inactivated fetal bovine serum and 1% penicillin/streptomycin. When the cells became confluent (~ 10 days), flasks were shaken for 1 h on an orbital shaker (220 rpm) at 37 °C to remove microglia. The flasks were then changed to a new medium and shaken overnight (~ 20 h). After a 1 h incubation of the collected medium at 37 °C on non-coated cell culture dishes, the non-adherent cells (OPCs) were re-plated at a density of 20,000 cells/cm^2^ in Neurobasal medium containing 2 mM glutamine, 1% penicillin/streptomycin, 10 ng/mL PDGF-AA, 10 ng/mL FGF-2, and 2% B27 supplement onto poly-DL-ornithine-coated plates. To differentiate the OPCs into mature OLGs, the culture medium was replaced with DMEM containing 1% penicillin/streptomycin, 10 ng/mL CNTF, 15 nM T3, and 2% B27 supplement. For chronological assessment of each cell marker’s expression during differentiation, the cells were fixed or collected at different time points indicated by the day from differentiation induction and then further analyzed.

### Preparation of recombinant human α-syn pre-formed fibrils (PFFs)

The purification of recombinant human α-syn was conducted in accordance with previously established method [8, 12, 20]. Briefly, human wild-type α-syn cDNA was cloned into the bacterial expression vector pRK172. Transformations and selection were performed using *E. coli* BL-21 (DE3) competent cells (BioDynamics) and ampicillin (100 μg/mL) in Luria-Bertani media. Following the overnight incubation of the transformed cells in Luria-Bertani media containing ampicillin (100 μg/mL) at 37 °C, the culture was incubated for another 5 h after a 300-fold dilution and then induced with 1 mM isopropyl-β-D-thiogalactopyranoside for 5 h at 37 °C. Bacterial pellets were then resuspended in high-salt buffer (1 M Tris-HCl, pH 7.5, and 1 mM EDTA), heated to 100 °C for 5 min, and centrifuged at 15,000 rpm for 15 min. The supernatants were subjected to chromatography on a Q-Sepharose fast-flow column (GE healthcare) with a gradient of 0 to 0.5 M NaCl in Tris buffer. Resulting proteins were dialyzed overnight against 50 mM Tris-HCl, 150 mM KCl, and pH 7.5 and centrifuged at 55,000 rpm at 4 °C for 20 min. The removal of endotoxin was performed with EndoTrap HD (800,053, Hyglos), and the concentration of lipopolysaccharide was confirmed to be less than < 0.035 EU/μg αS protein using the LAL endotoxin assay kit (L00350C, GenScript). For PFF generation, proteins were incubated with constant agitation at 37 °C for 3–7 days.

### Application of α-syn PFFs to primary oligodendroglial cell culture

**Fig. 3:**
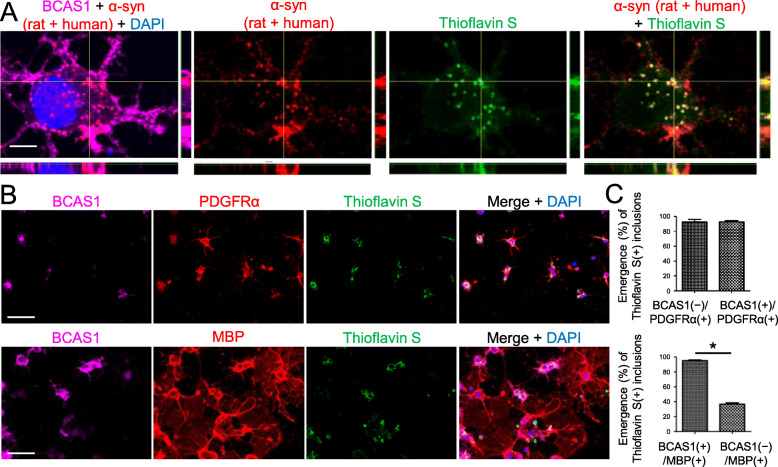
Extracellularly applied recombinant human α-syn PFFs induced cytoplasmic α-syn-immunoreactive inclusions in primary BCAS1(+) cell cultures. **a** Confocal images of BCAS1(+) cells, which were incubated with 1 μM α-syn PFFs for 24 h from day 4 after differentiation induction, showing the intracellular inclusions labeled with both anti-α-syn antibody and thioflavin S. Scale bar = 5 μm. **b** Immunostaining of oligodendroglial cells incubated with 1 μM α-syn PFFs for 24 h from days 3 (upper) and 4 (lower) after differentiation induction showing the ubiquitous development of thioflavin S-labeled inclusions in PDGFRα(+) cells and BCAS1(+) cells. In contrast, few BCAS1(−)/MBP(+) cells developed thioflavin S-labeled inclusions. Scale bar = 50 μm. **c** The percentages of oligodendroglial cells containing thioflavin S-labeled inclusions were compared between BCAS1(−)/PDGFRα(+) cells and BCAS1(+)/PDGFRα(+) cells (upper, performed on day 3), and between BCAS1(+)/MBP(+) cells and BCAS1(−)/MBP(+) cells (lower, performed on day 4). *N* = 4, respectively, independent culture, Mann–Whitney, p* < 0.05

**Fig. 4:**
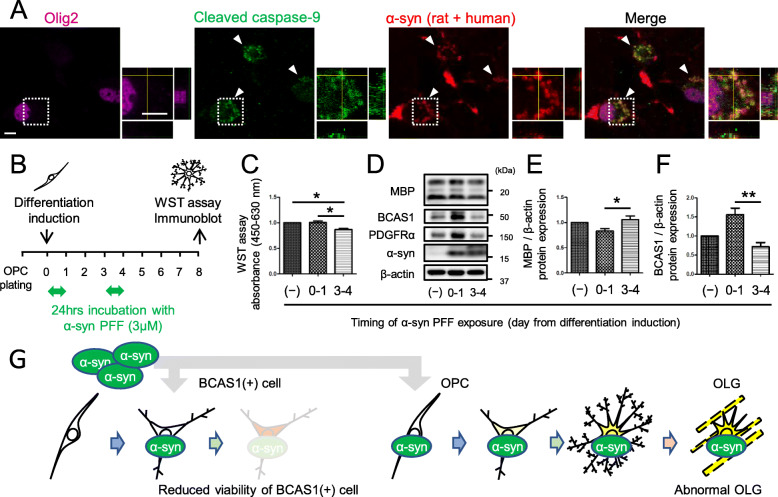
α-Syn PFF exposure to immature OLGs during differentiation-induced abnormal oligodendrogenesis. **a** Confocal images of oligodendroglial cells on day 4 after differentiation induction showing the colocalization of α-syn immunoreactivity and cleaved caspase-9 expression. The cells were incubated with 1 μM α-syn PFFs for 24 h (day 3–4) before fixation. White arrowheads indicate intracellular inclusions. The regions marked by dotted squares are highlighted in the magnified views. Scale bar = 5 μm. **b** Scheme showing the experimental protocols of 3 μM α-syn PFF exposure to oligodendroglial cells during two different phases (day 0–1, or day 3–4) of differentiation. **c** Cell viability analysis (WST assay) of day 8 OLGs, which were obtained through different protocols of α-syn PFF exposure during differentiation (no exposure, exposure on day 0–1, or exposure on days 3–4). *N* = 5, respectively, independent culture, Kruskal–Wallis, p* < 0.05. **d** Immunoblot analysis of day 8 OLGs obtained via different protocols of α-syn PFF exposure during differentiation (no exposure, exposure on day 0–1, or exposure on days 3–4), showing the protein expression levels of oligodendroglial cell markers. **e**, **f** Quantification of the relative protein expression levels of MBP (**e**) and BCAS1 (**f**) in day 8 OLGs obtained using the above-mentioned protocols. The differences between the no exposure group and day 0–1 group in (E) (*p* = 0.0636) and in (F) (*p* = 0.1980) were not statistically significant. *N* = 5, respectively, independent culture, Kruskal–Wallis, p* < 0.05, p** < 0.01. **g** Graphical representation of in vitro analysis showing two patterns of α-syn PFF-induced maturation inhibition. α-Syn PFF exposure to differentiating OLGs during the BCAS1(+) cell-dominant phase resulted in decreased cell viability (left), while exposure during the OPC-dominant phase resulted in abnormal protein expression levels of OLG markers (right)

To observe intracellular inclusions in OLG lineage cells (Fig. 3, Fig. 4a, Additional file 5 Fig. S4A), α-syn PFFs were diluted in PBS at 1 μM, sonicated several times (60 s in total), and diluted in media. Protein concentrations were determined using the bicinchoninic acid protein assay (Thermo Fisher), with bovine serum albumin as the standard. To evaluate the cell viability and the maturation of differentiating OLG lineage cells exposed to pathological α-syn (Fig. 4b–f), 3 μM α-syn PFFs was added to the culture medium at different time points (day 0–1 or day 3–4 from differentiation induction) and incubated for 24 h. After incubating with α-syn PFFs, cells were washed with DMEM containing 1% penicillin/streptomycin once to remove residual α-syn PFFs. The cells were then incubated with α-syn-free differentiation medium until day 8, at which point they were subject to the WST assay and immunoblot analysis.

### Protein labeling and time-lapse imaging

After sonication, α-syn PFFs (2 mg/mL) were labeled with the Alexa Fluor 594 protein labeling kit (Thermo Fisher, A10239) according to the manufacturer’s instructions. Time-lapse imaging was performed with a BZ-X710 camera (Keyence) equipped with an incubator (37 °C, 5% CO2), and images were acquired at defined positions every 10 min. The images were then converted to mp4 files. Immediately after time-lapse imaging, cells were fixed and subjected to immunostaining to validate the maturation status of the imaged cells (Additional file 5: Fig. S4B, C).

### Slice culture

Rat cortical slices were prepared from Sprague Dawley rats (Shimizu Laboratory Supplies Co., Ltd) on the 5th day after birth. The brains were removed and immediately submerged in cold HBSS with 0.6% D-glucose and 1% penicillin/streptomycin. Coronal slices from the cortices (100 μm) were cut using a vibrating-blade microtome (Neo-LinearSlicer, D.S.K). Slices were placed onto a 0.4 μm culture plate insert (Millicell-CM, Merck Millipore) and incubated for 24 h in six-well culture dishes containing 3 μM α-syn PFFs in 1.5 mL of DMEM containing 2% B27 supplement and 1% penicillin/streptomycin.

### Lysotracker

For the use of the LysoTracker (Life technologies) probes, samples were incubated with probe-containing medium (50 nM) for 30 to 60 min before fixation. Immunostaining was conducted as described above.

### WST assay

Cell viability was assessed by the WST reduction assay kit (Cell Counting Kit-8, Dojindo). Briefly, the cells were incubated in a 10% WST solution for 1 h at 37 °C. The absorbance of the culture medium was measured at a wavelength of 450 nm and a reference wavelength of 630 nm.

### Immunoblot analysis

For immunoblot analysis, cells were rinsed twice with PBS and collected into sample buffer containing 50% Tris-Glycine SDS buffer (Novex), 45% RIPA buffer (20 mM HEPES-KOH pH 7.4, 150 mM NaCl, 2 mM EDTA, 1% Nonidet-P40, and 1% sodium deoxycholate), 5% 2-mercaptomethanol (Nacalai tesque), 1% phosphatase inhibitor (Nacalai tesque), and 1% protease inhibitor (Nacalai tesque). Subsequently, the samples were heated at 95 °C for 5 min, and each sample was loaded onto a 5–20% polyacrylamide gel (Wako). After electrophoresis and the transfer of the gels onto PVDF membranes (Merck Millipore), the membranes were fixed with 4%PFA for 30 min and blocked in Tris-buffered saline containing 0.1% Tween 20 and 5% nonfat dry milk for 30–60 min at room temperature. The membranes were then incubated overnight at 4 °C with primary antibodies for MBP (1:500, Thermo Fisher Scientific, MA1–10837), BCAS1 (1:1000, Bioss antibodies, bs-11462R), α-syn (1:1000, BD Biosciences, 610,787), PDGFRα (1:500, Santa Cruz, sc-338), or anti-β-actin (1:10000, Sigma Aldrich, A5441). This was followed by a 60 min incubation with the appropriate secondary antibodies (Novus, NB7574 and NB7160) at room temperature, with visualization by enhanced chemiluminescence (Nacalai tesque). The density of each band was quantified using Image J software.

### Quantitative real-time PCR

For quantitative real-time PCR, RNA was extracted from the cells with RLT lysis buffer (QIAGEN) according to the manufacturer’s instructions. RNA concentration was measured using a NanoDrop 1000 spectrometer (Thermo Scientific). cDNA was generated using reverse transcription and the PrimeScript RT reagent kit (TaKaRa). The amount of cDNA was quantified with real-time PCR using the LightCycler 480 SYBR Green I Master mix (Roche) and a Roche LightCycler 480 system. The primer sets used in this study were as follows: *Pdgfrα*, forward: CTAATTCACATTCGGGAAGGTTG, reverse: GGACGATGGGCGACTAGAC, *Bcas1*, forward: AGGGAAGGACATAGTGGACAGC, reverse: TTCTCGGCGTCCGTGTCTT, *Mbp*, forward: ACACACAAGAACTACCCACTACGG, reverse: AGCTAAATCTGCTGAGGGACAG, *Snca*, forward: CAACAGTGGCTGAGAAGACC, reverse: GAAGGCATTTCATAAGCCTC.

### Statistical analysis

All quantitative data were analyzed using Prism 6.0 (Graphpad). The statistical significance was evaluated using Mann-Whitney’s U test for two-group comparisons or one-way non-parametric ANOVA (Kruskal-Wallis test) for multiple-group comparisons. Data are expressed as the mean ± S.D. Spearman rank correlation coefficient analysis was used for the correlation analysis of phosphorylated α-syn-immunoreactive areas and late-stage BCAS1(+) cell counts.

## Results

### Significant numbers of BCAS1-immunopositive cells were observed in the frontal cortices of MSA brains and aged control brains

Immunohistochemistry was performed using brain tissues from the frontal lobe (cortex and white matter), pons, cerebellum, and putamen of MSA patients and aged non-neurodegenerative disease controls (frontal lobe; MSA, *N* = 9, control, *N* = 6, cerebellum, pons, putamen; MSA, *N* = 4, control, *N* = 2). As previously described, significant numbers of BCAS1(+) cells were observed in the frontal cortex of both MSA and control brains, whereas the numbers are very limited in the white matter, cerebellum, pons, and putamen in both groups (Fig. 1a, Additional file 3: Fig. S2A) [6]. Accordingly, the numbers of BCAS1(+) cells in these regions were limited in both MSA-C and MSA-P cases (case 1, 2, 3, and 6, Additional file 1: Table S1), and it was difficult to perform statistical analysis regarding the difference in the numbers and morphological alteration of BCAS1(+) cells between the MSA subtypes. In the frontal cortex, compared with control brains, a trend showing an increase in BCAS1(+) cell counts and a decrease in MBP-immunoreactive area was observed in MSA brains; however, these differences were not statistically significant (Fig. 1 A–C). Phosphorylated α-synuclein (p-α-syn)-immunoreactive inclusions were observed with varying frequencies and were accompanied by a significant reduction in NeuN(+) neuronal cell counts (Fig. 1 A, D, E).

### Phosphorylated α-synuclein density negatively correlates with the numbers of BCAS1(+) cells in mature morphology in the frontal cortex of MSA brains

On the basis of the observation that there is a population of differentiating OLGs immunolabeled with BCAS1 predominantly in the frontal cortex, we next focused on the maturation of BCAS1(+) cells of this region in the presence of p-α-syn-immunoreactive inclusions. Consistent with a previous report, BCAS1(+) cells were not immunolabeled with other oligodendroglial cell markers such as CC1 and NG2 in adult human brains (Additional file 3 Fig. S2B) [6]. Given these characteristics of differentiating OLGs, BCAS1(+) cells were morphologically classified into two groups (Fig. 1f). As such, BCAS1(+) cells displaying OPC-like morphology (three or less visible processes) were classified as early-stage BCAS1(+) cells (Fig. 1 Ga, b), whereas those displaying a mature OLG-like morphology (four or more visible processes) and/or myelinating morphology were classified as late-stage BCAS1(+) cells (Fig. 1 gc, d). Spearman rank correlation analysis revealed a negative correlation between the numbers of late-stage BCAS1(+) cells and the density of p-α-syn(+) area in the frontal cortices of MSA patients (*N* = 9, *r* = − 0.8000, *p* = 0.0138) (Fig. 1h, i). This correlation still existed when analysis was conducted only with MSA-C cases (*N* = 7, *r* = − 0.7857, *p* = 0.0480, data not shown). However, a correlation was not observed in the frontal cortex of PD/DLB patients (*N* = 9, *r* = 0.2857, *p* = 0.5560) or in the corresponding white matter regions of MSA patients (*N* = 9, *r* = − 0.1000, *p* = 0.8100) (Additional file 3: Fig. S2A, C, D). As for BCAS1(+) cells in the white matter, only limited numbers of the total BCAS1(+) cells were present irrespective of the disease type, which is in line with the previous observation by Fard MK et al. [6]. Therefore, there was no obvious correlation between the numbers of early-stage BCAS1(+) cells and the density of p-α-syn immunoreactivity (Additional file 3: Fig. S2E).

### Phosphorylated α-synuclein-immunoreactive inclusions were observed in some BCAS1(+) cells of MSA patient brains

Based on the observation of BCAS1(+) cells in the frontal cortex, we then asked how p-α-syn pathology affects BCAS1(+) cell maturation in MSA brains. To address this question, we investigated the presence of cytoplasmic inclusions in BCAS1(+) cells. Brain tissues from cases with evident cortical p-α-syn pathology (MSA; *N* = 4 PD/DLB; *N* = 7, “stage 2” or greater cortical α-syn pathology, Additional file 1: Table S1) were analyzed via confocal microscopy in conjunction with immunofluorescence staining. Among these cases, the numbers of late-stage BCAS1(+) cells, but not those of early-stage BCAS1(+) cells, in the MSA brains were found to be significantly decreased compared with controls and PD/DLB cases (Additional file 3: Fig. S2F, G). In addition, the ratio of late-stage BCAS1(+) cells to total BCAS1(+) cells was also decreased in MSA patient tissues compared with controls (Additional file 3: Fig. S2H). Cytoplasmic inclusions labeled with the anti-p-α-syn antibody were detected within the BCAS1(+) cell population present primarily in the deep cortical layer of the MSA brains (Fig. 2a). These BCAS1(+) cell inclusions were found exclusively among MSA cases (Fig. 2b–d). Morphologically, the frequency of cytoplasmic inclusions within the BCAS1(+) cells of the MSA cases was significantly higher for early-stage BCAS1(+) cells than for late-stage BCAS1(+) cells (Fig. 2e, Additional file 3: Fig. S2I). The majority of the inclusions within BCAS1(+) cells were immunoreactive toward the anti-cleaved caspase-9 antibody, as previously reported for GCIs (Fig. 2b, f) [9]. These inclusions in BCAS1 (+) cells were observed not only in the frontal cortices but also in the pons of MSA patients (Additional file 3: Fig. S2J).

### Primary BCAS1(+) cells were observed to incorporate extracellular α-syn PFFs

Given that some differentiating BCAS1(+) cells in MSA brains contained p-α-syn-immunoreactive inclusions, we next examined the in vitro uptake of extracellular α-syn PFFs in primary oligodendroglial cell cultures. Consistent with previous studies, chronological analysis of the differentiating oligodendroglial cells showed a noticeable change in the morphology and protein expression of oligodendroglial cell markers (PDGFRα, BCAS1, MBP, NG2, and CC1) as well as in the mRNA expressions of these markers and *SNCA* (Additional file 4: Fig. S3A–D) [6, 17]. The highest mRNA expression of BCAS1 was observed on day 2 after differentiation induction, whereas a drastic increase in BCAS1 protein expression was observed from day 1 to 3 (Additional file 4: Fig. S3E–G). Given these findings, oligodendroglial cells were incubated with α-syn PFFs for 24 h from day 3 or day 4 after differentiation induction. Confocal microscopy revealed cytoplasmic inclusions within BCAS1(+) cells that were stained with both thioflavin S and anti-α-syn antibody (Fig. 3a). Double labeling with LysoTracker and anti-human α-syn antibody confirmed the internalization of the extracellularly applied α-syn PFFs (Additional file 5: Fig. S4A). To quantify α-syn PFF uptake at each stage of oligodendroglial differentiation, the emergence of thioflavin S-labeled cytoplasmic inclusions was compared between BCAS1(−)/PDGFRα(+) cells and BCAS1(+)/PDGFRα(+) cells as well as between BCAS1(+)/MBP(+) cells and BCAS1(−)/MBP(+) cells. Based on the results from the chronological assessment of cell marker expression via immunostaining (Additional file 4: Fig. S3A), 24-h α-syn PFF exposure to differentiating OLGs was initiated on day 3 for the comparison between BCAS1(−)/PDGFRα(+) cells and BCAS1(+)/PDGFRα(+) cells and on day 4 for the comparison between BCAS1(+)/MBP(+) cells and BCAS1(−)/MBP(+) cells to ensure comparable numbers of cells in each group. Our results indicated that inclusions were detected in PDGFRα(+) cells regardless of the presence of BCAS1 expression (Fig. 3b, c). In contrast, inclusions were only sparsely observed in MBP(+) cells when BCAS1 expression was diminished (Fig. 3b, c). This maturation-dependent reduction in the frequency of inclusion formation was also visualized through the time-lapse imaging of each oligodendroglial cell treated with Alexa Fluor 594-labeled α-syn PFFs and sequential immunolabeling of the cells immediately after time-lapse imaging (Additional files 4, 5, 6: Supplementary Video 1–3, Additional file 5: Fig. S4B, C). Slice cultures of rat brains further confirmed that the uptake of α-syn PFFs into PDGFRα(+) cells and BCAS1(+) cells occurred in an ex vivo setting (Additional file 5: Fig. S4D).

### Exposure of α-syn PFFs during the immature state of OLGs can result in both reduced cell viability and insufficient maturation of OLGs

Interestingly, α-syn-immunopositive inclusions within the differentiating oligodendrocytes, which were exposed with α-syn PFFs for 24 h (days 3–4), were colocalized with cleaved caspase-9 (Fig. 4a). On this basis, the impacts of transient α-syn PFFs exposure on cell viability and OLG maturation during differentiation were analyzed and compared between the two α-syn PFF exposure protocols. The first protocol involved exposure during the PDGFRα(+) cell-dominant phase (on days 0–1 after differentiation induction), whereas the second protocol involved exposure during the BCAS1(+) cell-dominant phase (on days 3–4 after differentiation induction) (Fig. 4b). α-Syn PFF exposure on days 3–4 after differentiation induction resulted in reduced cell viability, as measured by the WST assay, compared with OLGs without α-syn PFF exposure and those with α-syn PFF exposure on days 0–1 after differentiation induction (Fig. 4c). In contrast, α-Syn PFF exposure on days 0–1 after differentiation induction caused lower MBP expression and higher BCAS1 expression as compared with exposure on days 3–4 (Fig. 4d–f).

## Discussion

BCAS1 is a marker for distinctive oligodendroglial cell population undergoing differentiation. The abundance of BCAS1(+) cells indicates the presence of active OLG generation [6]. Nevertheless, numerous questions regarding BCAS1(+) cells remain to be answered, such as what percentage of BCAS1(+) cells successfully differentiate into myelinating OLGs, and whether or how BCAS1(+) cells play a role in brain homeostasis. In the present study, the unique characteristics of BCAS1 expression in the ongoing process of OLG maturation, which could be immunohistochemically visualized, enabled us to elucidate impaired BCAS1(+) cell differentiation in a p-α-syn density-dependent manner (Fig. 1h, i). BCAS1 is a useful marker to interpret the maturation status of OLGs particularly in the frontal cortex of adult human brains. However, BCAS1(+) cells are sparsely distributed in the pons, cerebellum, and putamen, which are the regions frequently affected by α-syn pathology in MSA (Additional file 3: Fig. S2A). On the other hand, we quantified the numbers of BCAS1(+) cells in the white matter where the numbers of OLGs should be more than that present in the cortex. As expected, the numbers of p-α-syn-immunoreactive inclusions in the white matter were significantly higher than those in the cortex (white matter; 190.8 ± 57.5 /1.57 mm^2^, cortex; 27.6 ± 4.7 /1.57 mm^2^, *N* = 9, Mann–Whitney, *p* = 0.0028). However, the numbers of BCAS1(+) cells in the white matter were very limited, which is in accordance with the previous report by Fard MK et al. [6]. It is also possible that the morphological differences in BCAS1(+) cells may be less detectable between regions with excessive p-α-syn accumulation. Such differences in distribution and morphology in BCAS1(+) cells in the different areas (i.e., frontal cortex/white matter, pons, cerebellum, and putamen) may be attributable to the regional heterogeneity of OLG lineage cells [11].

The limitation of the present study is that only four MSA cases showed evident p-α-syn accumulation in the frontal cortex (Additional file 1: Table S1). The average disease duration of these four cases (one MSA-P and three MSA-C cases) is relatively longer than that of other MSA cases (6.0 ± 1.9 vs 4.5 ± 0.8 years, Mann–Whitney, *p* = 0.7714). Therefore, the present study of inclusions in BCAS1(+) cells in the frontal cortex may represent pathological alterations in advanced stage of MSA. Another limitation is that our MSA cohort includes cases with two clinical subtypes: MSA-P (*N* = 2) and MSA-C (*N* = 7). Although the data from MSA-P cases are largely comparable with those from MSA-C cases, there is a possibility that our statistical analysis represents the conditions in MSA-C.

Myelin dysfunction has not been reported as a main pathological feature in PD/DLB. In fact, our post-mortem study of PD/DLB cases did not reveal either a significant reduction in the numbers of late-stage BCAS1(+) cells (Additional file 3: Fig. S2F, H) or in the presence of cytoplasmic inclusions in BCAS1(+) cells (Fig. 2 C, D). It is likely that immature OLGs in PD/DLB brains are not affected as strikingly as those in MSA brains, although it is uncertain whether these findings reflect the different pathogenicities of disease-specific α-syn strains in MSA and PD/DLB.

Increasing evidence supports the hypothesis that the cell-to-cell, prion-like propagation of misfolded α-syn results in MSA disease progression [15, 22]. However, it is still enigmatic how α-syn pathology spreads almost exclusively among the OLGs in MSA brains [7]. Our study also suggests that, at least in some cases, the OLG lineage cell-specific inclusion pathology in MSA brains can be observed during BCAS1(+) phase. Intracerebral injection of MSA brain homogenates into mouse results in the formation of inclusions predominantly in the neurons, indicating that MSA-specific α-syn strain does not solely contribute to the OLG-specific distribution of the inclusions [2]. These data raise a possibility that the propagation of the GCI pathology in MSA requires some alteration in mature OLG function, which blocks the entry of pathogenic α-syn under normal conditions. A previous report suggested that an altered expression pattern of TPPP/p25α appears before the development of GCIs [18]. Our in vitro data suggest that BCAS1(−)/MBP(+) cells incorporate extracellular α-syn PFFs less effectively than BCAS1 (+)/MBP(+) cells (Fig. 3b, c). Taken together the drastic change in the reaction to the extracellular α-syn PFF may be of great importance, considering that the protective function of normal mature OLGs may be lost, resulting in vulnerability against the invasion of pathological α-syn in MSA. Because BCAS1(−)/PDGFRα(+) OPCs also take up extracellular α-syn PFFs, BCAS1 expression may not be an exclusive modulator of α-syn uptake.

Several studies have reported that myelin dysfunction precedes the loss of OLGs in MSA brains, suggesting the abundant presence of defective OLGs [4, 13]. On the other hand, the number of OPCs has been reported to be increased in MSA [1, 13]. Based on these observations, two hypotheses can be proposed for the presence of defective OLGs in MSA brains: (1) OLGs newly differentiated from OPCs are defective, thereby retaining some features of immature OLGs, and (2) defective OLGs cannot be replaced by healthy OLGs due to the death of differentiating oligodendroglial cells. Our in vitro data supports both scenarios; the burden of aggregated α-syn in BCAS1(−) OPCs leads to the insufficient maturation of OLGs, whereas that in BCAS1(+) cells leads to reduced cell viability and normal MBP expression (Fig. 4g). The mechanism of α-syn-induced cytotoxicity in BCAS1(+) cells may be mediated via caspase-9 activation, as previously suggested with GCIs in mature OLGs (Fig. 4a) [9]. However, our post-mortem analysis of human brains showed decreased numbers of late-stage BCAS1(+) cell and p-α-syn/cleaved caspase-9-immunoreactive inclusions in early-stage BCAS1(+) cells without significant increase in the total BCAS1(+) cell counts in MSA brains. These human data may support the scenario that late-stage BCAS1(+) cells cannot survive or early stage BCAS(-) cells cannot differentiate into late stage under the burden of pathological α-syn during differentiation. The contribution of p-α-syn accumulation in BCAS1(+) cells to GCI formation in MSA brains needs further investigations.

## Conclusions

The present study demonstrated that α-syn-immunoreactive inclusions occur in a proportion of immature differentiating OLGs and that α-syn disturbs the process of OLG regeneration during the early phase of differentiation in MSA brains. Although the reduced density of late-stage BCAS1(+) cells in MSA brains indicates α-syn-induced cytotoxicity against differentiating OLGs or the suppression of proper maturation, the presence of inclusions in BCAS1(+) cells suggests that some oligodendroglial pathology appears before complete OLG maturation. Overall, our novel findings provide direct evidence of impaired oligodendroglial maturation during the BCAS1(+) state in post-mortem MSA brains and indicate that reinforcement of oligodendroglial maturation can serve as a promising therapeutic strategy against neurodegeneration in MSA.

## Supplementary information

**Additional file 1: Table S1.** Clinical information for autopsy cases.

**Additional file 2: Figure S1.** Sampled regions of all studied cases in the present study.

**Additional file 3: Figure S2.** Extensive analysis of BCAS1(+) cells in MSA and PD/DLB brains.

**Additional file 4: Figure S3.** Characterization of oligodendroglial cell marker expressions during differentiation in primary oligodendroglial cell culture.

**Additional file 5: Figure S4.** In vitro inclusion formation in BCAS1(+) cells.

**Additional file 6: Movie S1.** Time-lapse video of primary OPC cultures incubated with Alexa Fluor 594-labeled α-syn PFFs.

**Additional file 7: Movie S2.** Time-lapse video of primary oligodendroglial cell cultures at 4 days of differentiation incubated with Alexa Fluor 594-labeled α-syn PFFs.

**Additional file 8: Movie S3.** Time-lapse video of primary OLG culture incubated with Alexa Fluor 594-labeled α-syn PFFs.

## Data Availability

All data generated or analyzed during this study are included in this published article.

## Abbreviations

α-syn: α-synuclein
BCAS1: Breast carcinoma-amplified sequence 1
DLB: Diffuse Lewy body disease
GCI: Glial cytoplasmic inclusion
MBP: Myelin basic protein
MSA: Multiple system atrophy
NeuN: Neuronal nuclei
NG2: Nerve-glia antigen 2
OLG: Oligodendrocyte
OPC: Oligodendrocyte precursor cell
p-α-syn: Phosphorylated α-synuclein
PD: Parkinson’s disease
PDGFRα: Platelet derived growth factor receptor α
PFF: Pre-formed fibril
