# Supplementary material for: BCAS1-positive immature oligodendrocytes are affected by the α-synuclein-induced pathology of multiple system atrophy
Source: Acta Neuropathol Commun. 2020 Jul 29;8:120. doi: 10.1186/s40478-020-00997-4 (PMC7391509; doi:10.1186/s40478-020-00997-4)
Supplement: Supplementary file 1 — Additional file 1: Table S1. Clinical information for autopsy cases. [file 40478_2020_997_MOESM1_ESM.docx]

Table S1. Clinical information for autopsy cases

| Case | Diagnosis | Age | Sex | Brain weight (g) | Disease duration (yrs) | PMI (hrs) | Cortical α-syn^*^ | Regions studied |
| --- | --- | --- | --- | --- | --- | --- | --- | --- |
| 1 | MSA-P | 52 | F | 1300 | 3 | 2.5 | 1 | FCx, FWM, Pu, Po, Ce |
| 2 | MSA-P | 72 | F | 1205 | 12 | 4 | 3 | FCx, FWM, Pu, Po, Ce |
| 3 | MSA-C | 60 | M | 940 | 6 | 2.5 | 3 | FCx, FWM, Pu, Po, Ce |
| 4 | MSA-C | 71 | F | 1180 | 4 | 8 | 2 | FCx, FWM |
| 5 | MSA-C | 78 | M | 1100 | 3 | 2 | 1 | FCx, FWM |
| 6 | MSA-C | 66 | F | 1020 | 7 | 1.5 | 1 | FCx, FWM, Pu, Po, Ce |
| 7 | MSA-C | 66 | M | 1360 | 4 | 0.5 | 1 | FCx, FWM |
| 8 | MSA-C | 72 | F | 990 | NA | 2 | 1 | FCx, FWM |
| 9 | MSA-C | 71 | M | 1105 | 2 | 3 | 3 | FCx, FWM |
| 10 | MSA-C | 76 | M | 1110 | 7 | 3 | NA | Po |
| Sub-total |  | 68.4 |  |  |  |  |  |  |
| 11 | PD | 79 | F | 1130 | 13 | 0.5 | 3 | FCx, FWM |
| 12 | PD | 76 | M | 1200 | 14 | 2.5 | 1 | FCx, FWM |
| 13 | PD | 81 | F | 1040 | Over 3 | 2.5 | 2 | FCx, FWM |
| 14 | PD | 88 | M | 1360 | Over 10 | 2 | 2 | FCx, FWM |
| 15 | PD | 78 | F | 1090 | 11 | 12 | 1 | FCx, FWM |
| 16 | DLB | 69 | M | NA | 9 | 12 | 4 | FCx, FWM |
| 17 | DLB | 69 | M | NA | 1 | 7.5 | 4 | FCx, FWM |
| 18 | DLB | 86 | M | 1250 | 2 | 5 | 3 | FCx, FWM |
| 19 | DLB | 77 | M | 1240 | 12 | 12 | 2 | FCx, FWM |
| Sub-total |  | 78.1 |  |  |  |  |  |  |
| 20 | Old CI | 86 | M | 1130 |  | 5 | 0 | FCx, FWM, Pu |
| 21 | Pontine hemorrhage | 83 | F | 1105 |  | 0.5 | 0 | FCx, FWM |
| 22 | Wegener granulomatosis | 74 | F | NA |  | NA | 0 | FCx, FWM |
| 23 | Renal failure | 49 | M | 1350 |  | 7 | 0 | FCx, FWM |
| 24 | Malignant melanoma | 68 | F | 1040 |  | 6 | 0 | FCx, FWM |
| 25 | Old CI | 74 | F | 1080 |  | 9.5 | 0 | FCx, FWM, Pu |
| Sub-total |  | 72.3 |  |  |  |  |  |  |
| Total |  | 72.8 |  |  |  |  |  |  |
