# Supplementary material for: BCAS1-positive immature oligodendrocytes are affected by the α-synuclein-induced pathology of multiple system atrophy
Source: Acta Neuropathol Commun. 2020 Jul 29;8:120. doi: 10.1186/s40478-020-00997-4 (PMC7391509; doi:10.1186/s40478-020-00997-4)
Supplement: Supplementary file 2 — Additional file 2: Figure S1. Sampled regions of all studied cases in the present study. [file 40478_2020_997_MOESM2_ESM.pdf]

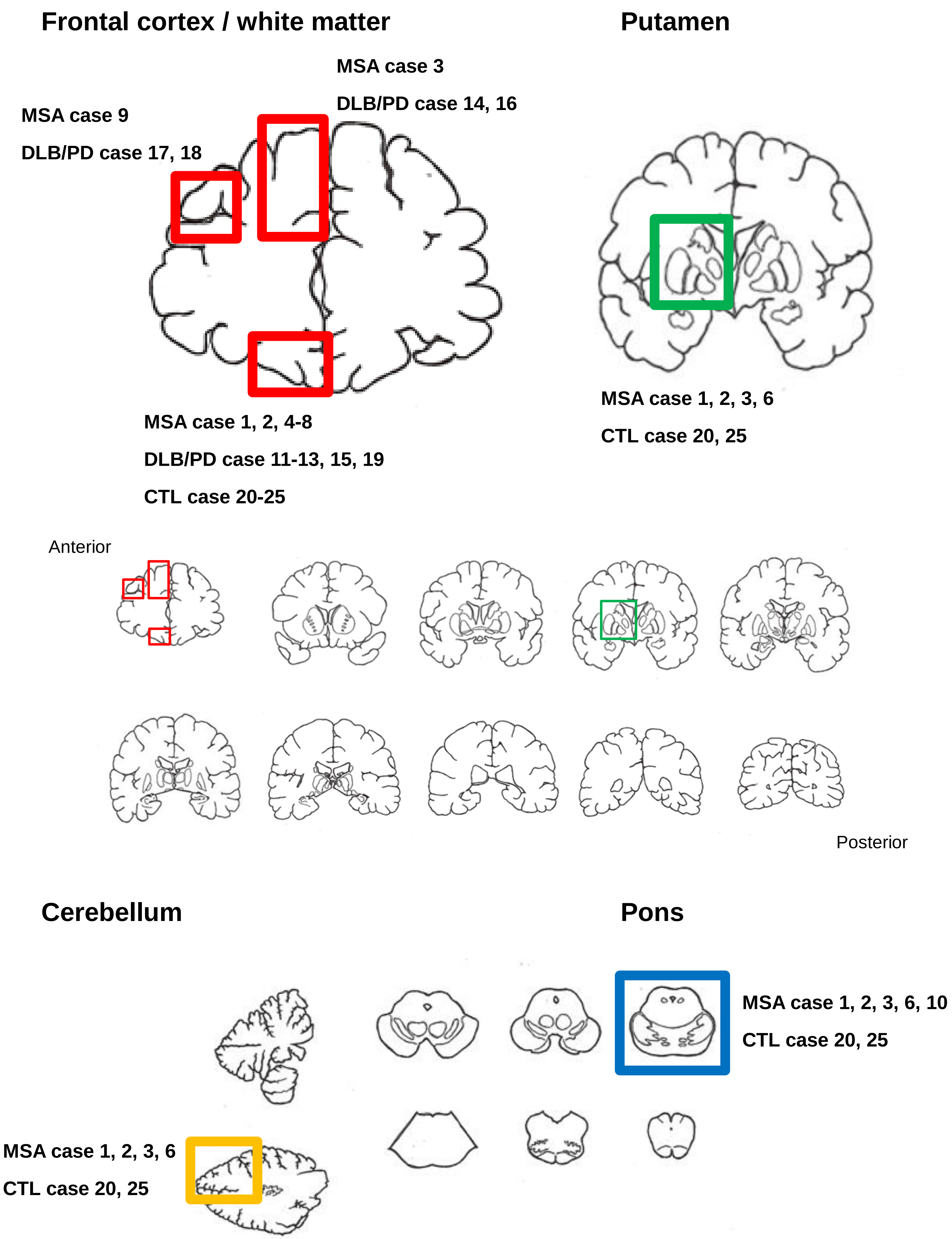
Frontal cortex / white matter
MSA case 3
DLB/PD case 14, 16
MSA case 9
DLB/PD case 17, 18
MSA case 1, 2, 4-8
DLB/PD case 11-13, 15, 19
CTL case 20-25
Putamen
MSA case 1, 2, 3, 6
CTL case 20, 25
Anterior
Posterior
Cerebellum
MSA case 1, 2, 3, 6
CTL case 20, 25
Pons
MSA case 1, 2, 3, 6, 10
CTL case 20, 25
