# Supplementary figures and images for: BCAS1-positive immature oligodendrocytes are affected by the α-synuclein-induced pathology of multiple system atrophy
Source: Acta Neuropathol Commun. 2020 Jul 29;8:120. doi: 10.1186/s40478-020-00997-4 (PMC7391509; doi:10.1186/s40478-020-00997-4)

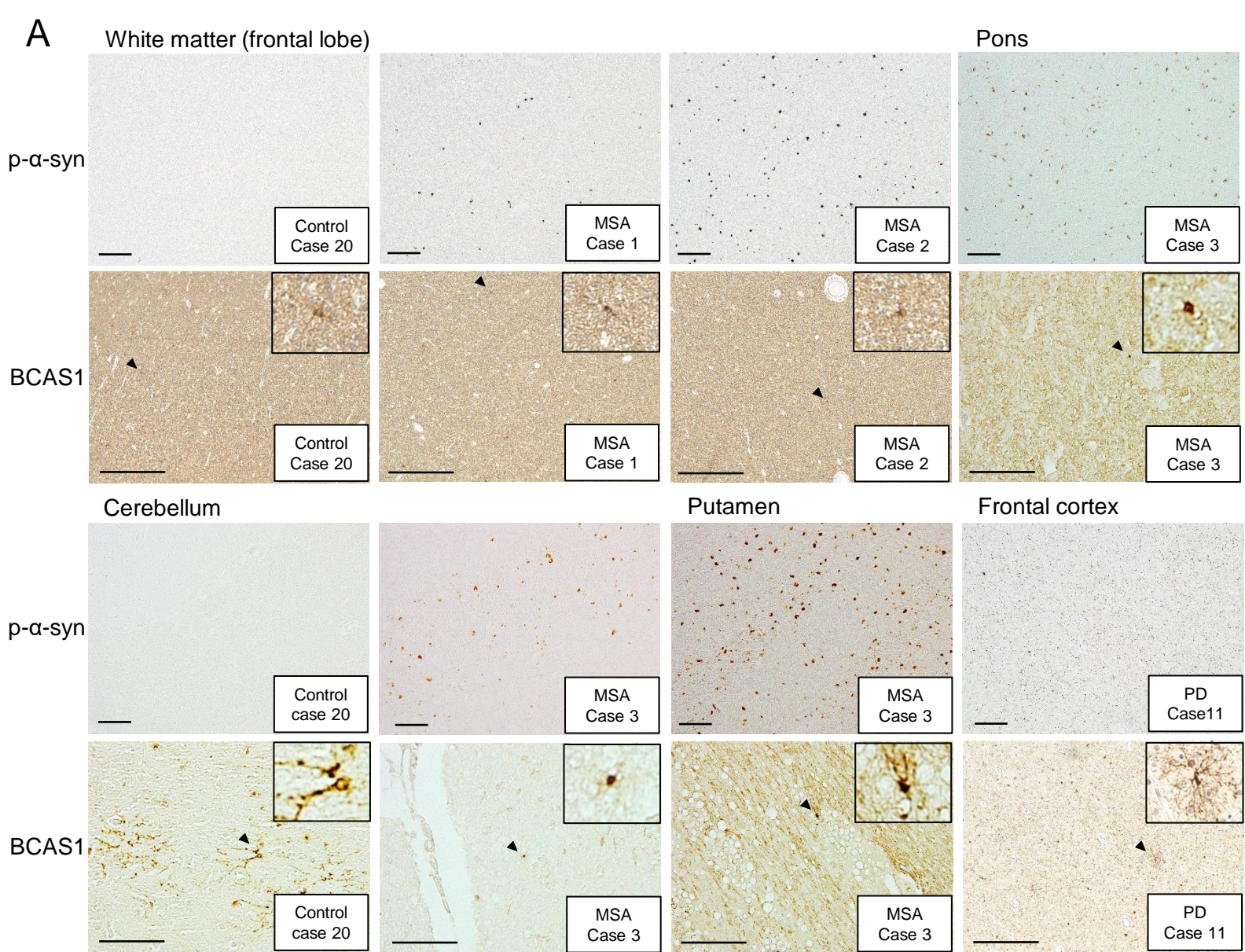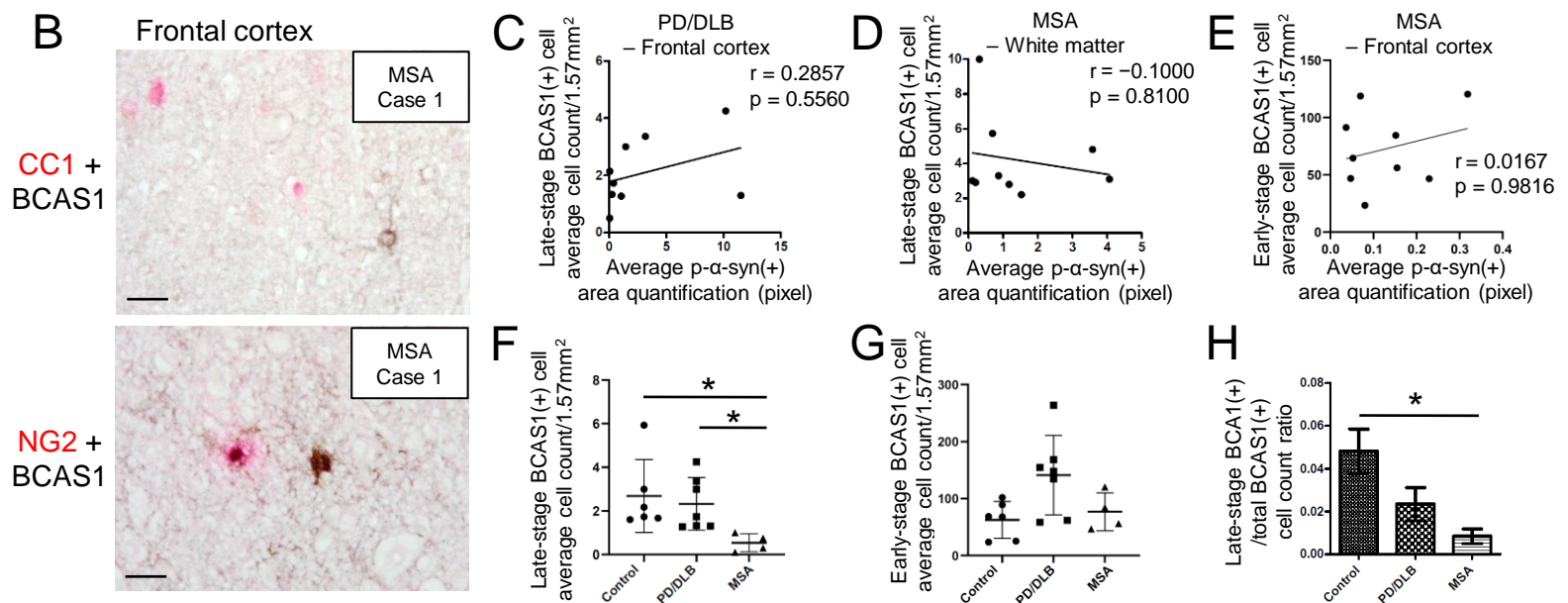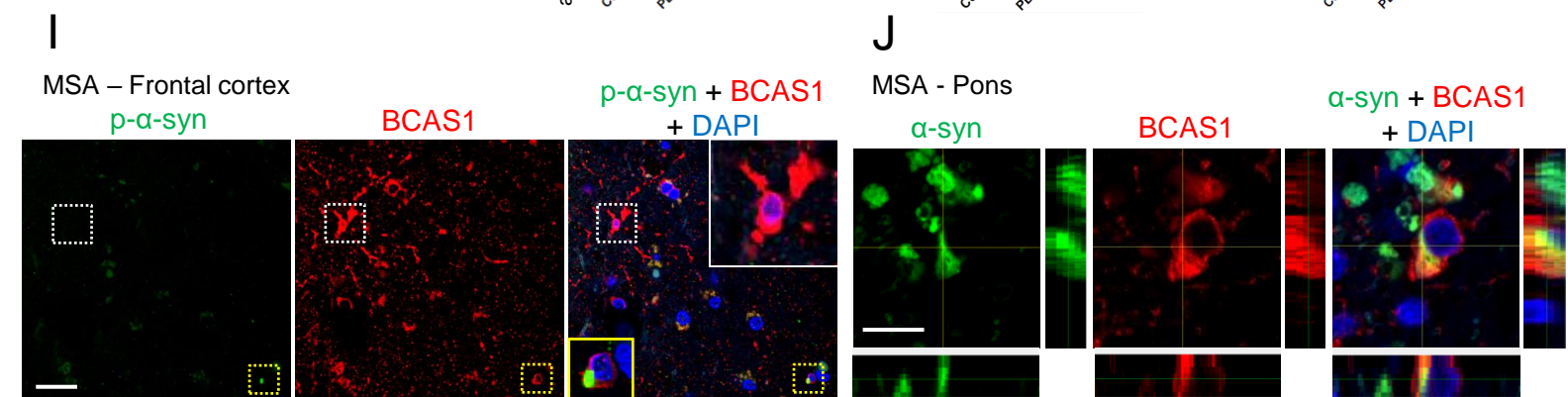

Supplement: Supplementary file 3 — Additional file 3: Figure S2. Extensive analysis of BCAS1(+) cells in MSA and PD/DLB brains. [file 40478_2020_997_MOESM3_ESM.pdf]

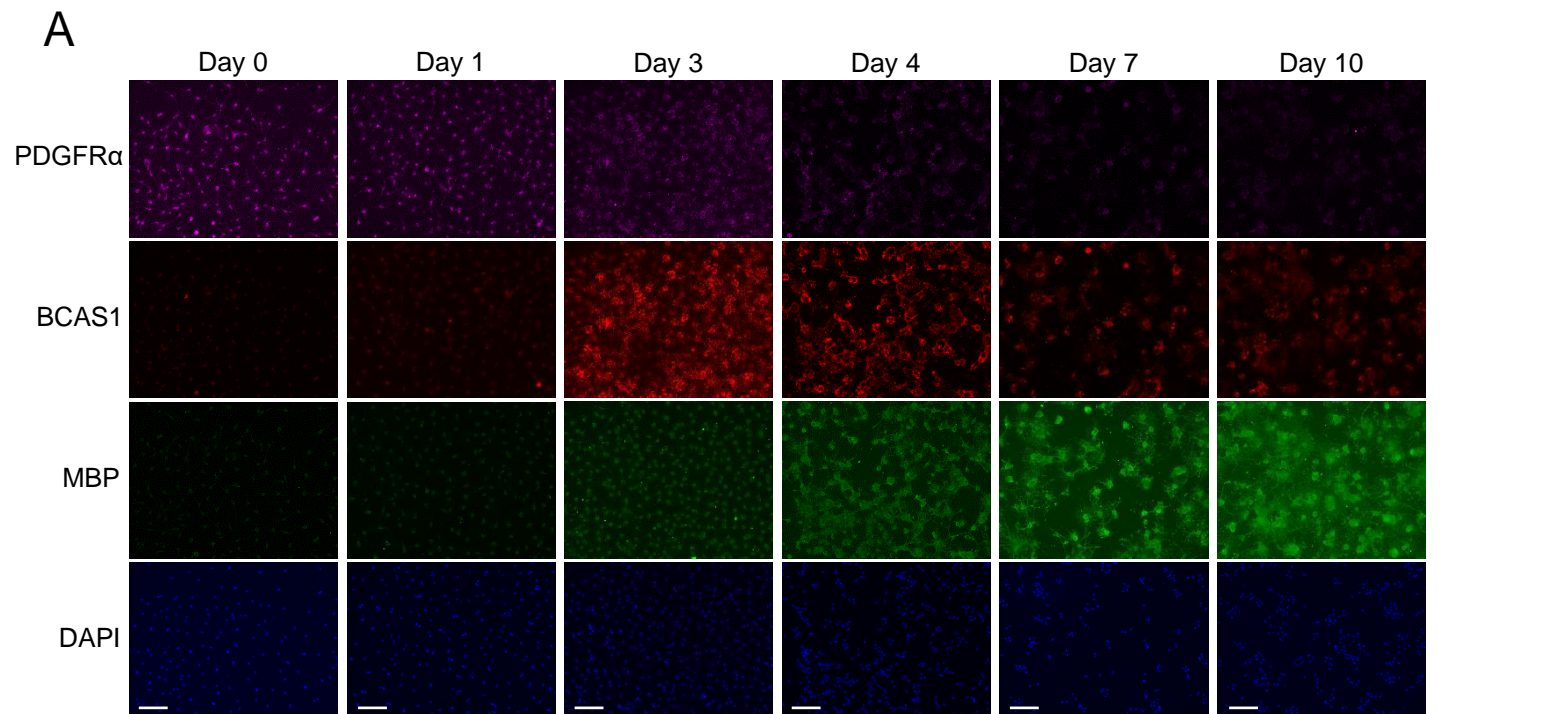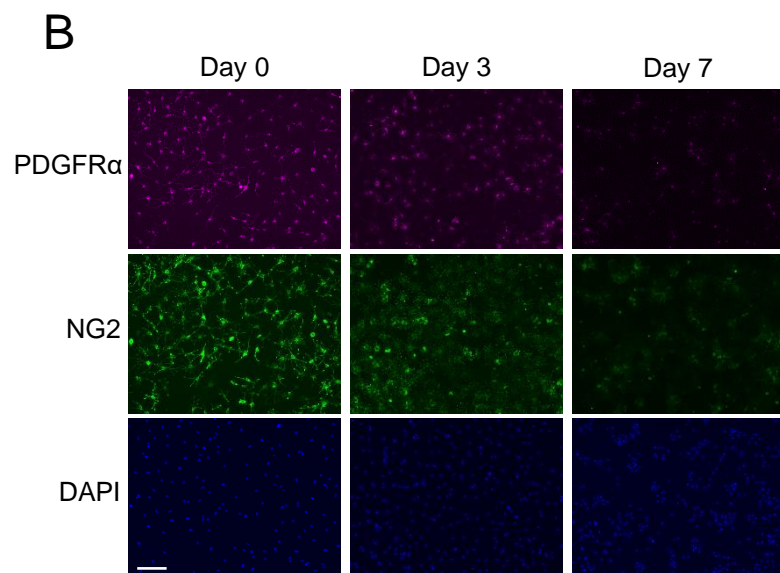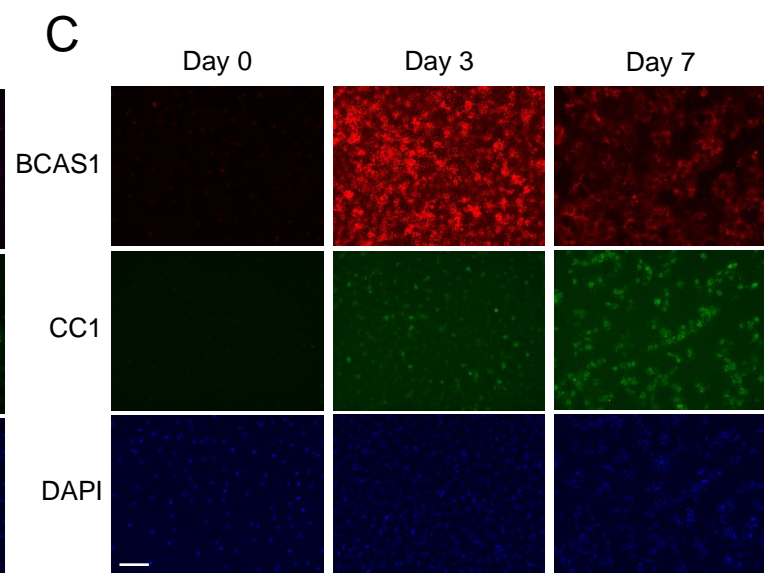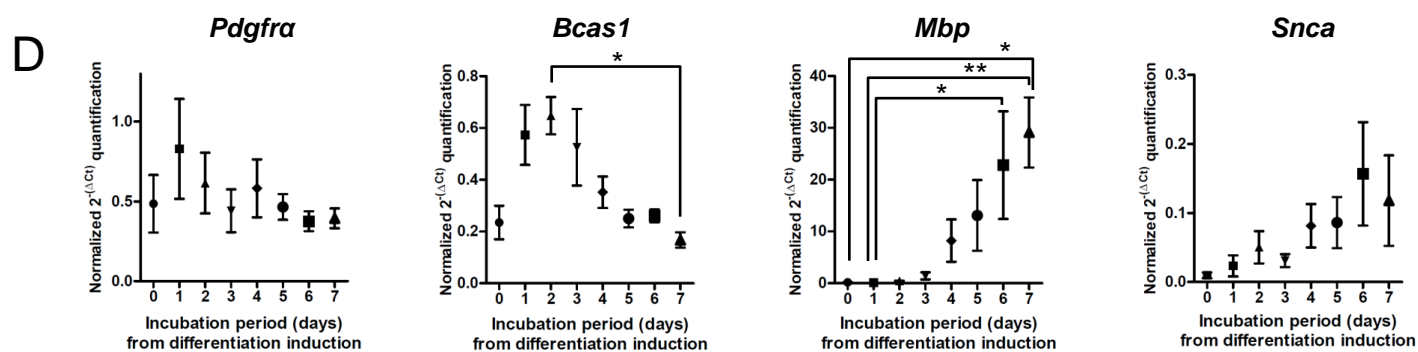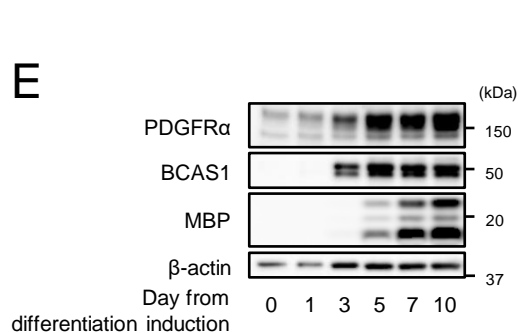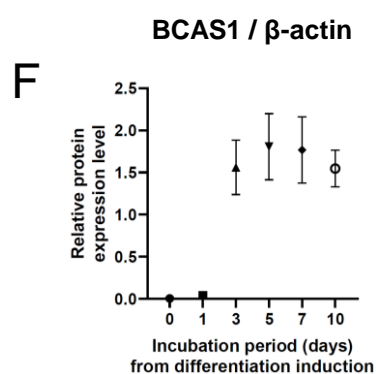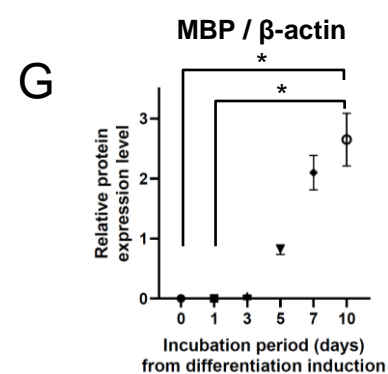

Supplement: Supplementary file 4 — Additional file 4: Figure S3. Characterization of oligodendroglial cell marker expressions during differentiation in primary oligodendroglial cell culture. [file 40478_2020_997_MOESM4_ESM.pdf]
