# Supplementary material for: BCAS1-positive immature oligodendrocytes are affected by the α-synuclein-induced pathology of multiple system atrophy
Source: Acta Neuropathol Commun. 2020 Jul 29;8:120. doi: 10.1186/s40478-020-00997-4 (PMC7391509; doi:10.1186/s40478-020-00997-4)
Supplement: Supplementary file 5 — Additional file 5: Figure S4. In vitro inclusion formation in BCAS1(+) cells. [file 40478_2020_997_MOESM5_ESM.pdf]

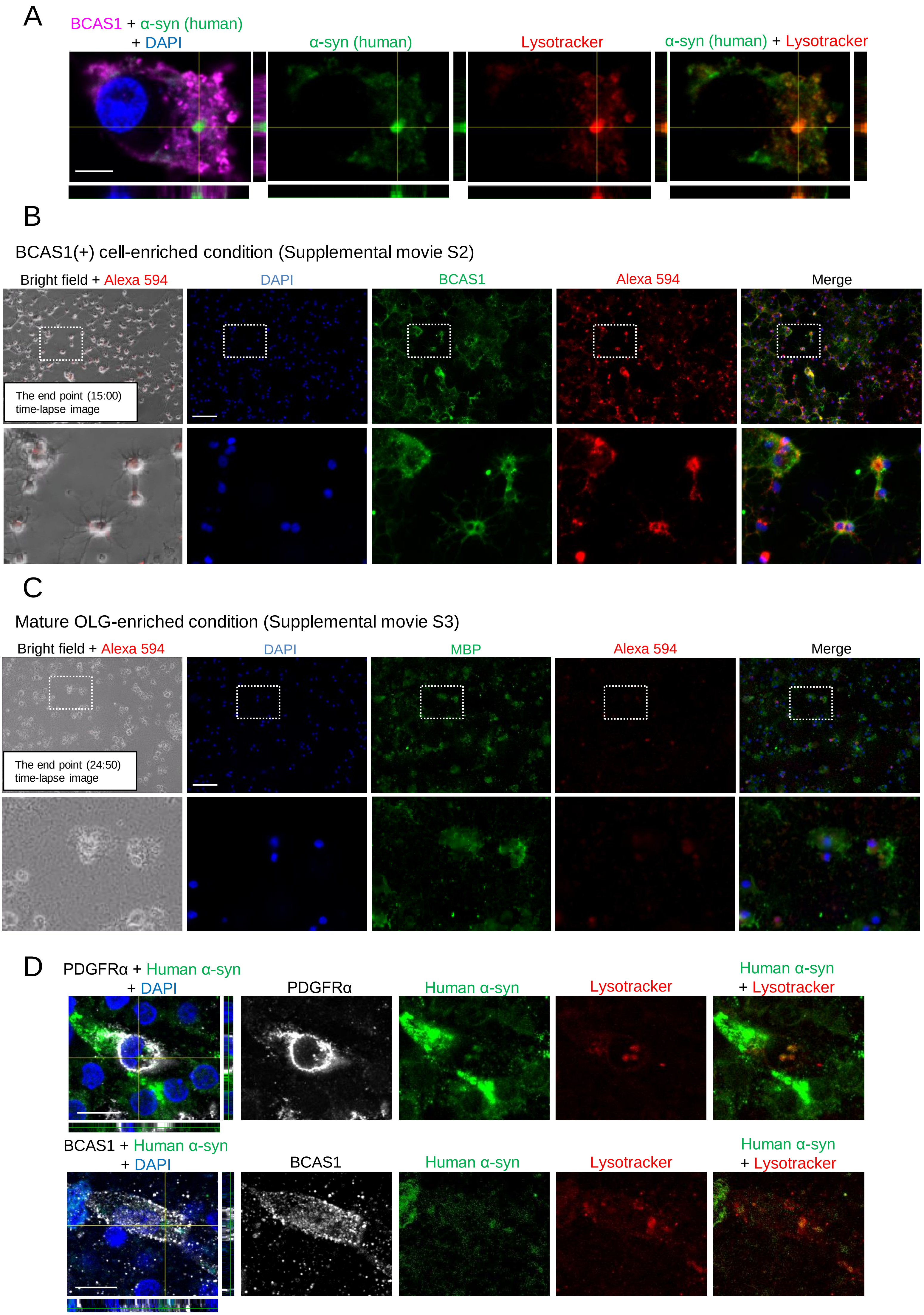
A
BCAS1 + α-syn (human) + DAPI
α-syn (human)
Lysotracker
α-syn (human) + Lysotracker
B
BCAS1(+) cell-enriched condition (Supplemental movie S2)
Bright field + Alexa 594
DAPI
BCAS1
Alexa 594
Merge
The end point (15:00) time-lapse image
C
Mature OLG-enriched condition (Supplemental movie S3)
Bright field + Alexa 594
DAPI
MBP
Alexa 594
Merge
The end point (24:50) time-lapse image
D
PDGFRα + Human α-syn + DAPI
PDGFRα
Human α-syn
Lysotracker
Human α-syn + Lysotracker
BCAS1 + Human α-syn + DAPI
BCAS1
Human α-syn
Lysotracker
Human α-syn + Lysotracker
